# Role of Adjuvant Radiosurgery after Thoracoscopic Microsurgical Resection of a Spinal Schwannoma

**DOI:** 10.1155/2012/345830

**Published:** 2012-07-18

**Authors:** Toba N. Niazi, Christian A. Bowers, Meic H. Schmidt

**Affiliations:** Department of Neurosurgery, Clinical Neurosciences Center, University of Utah, 175 N. Medical Drive East, Salt Lake City, UT 84132, USA

## Abstract

Stereotactic radiosurgery to benign tumors of the spine has not been advocated as a primary treatment modality because of the favorable prognosis for these lesions after gross-total resection. There is even less evidence regarding its use as an adjuvant to neurosurgical resection of benign recurrent spinal disease. We describe the case of a 30-year-old man with a thoracic spinal schwannoma who had an interval increase of his lesion five months after thoracoscopic microsurgical resection. The patient opted for noninvasive stereotactic radiosurgery in lieu of additional surgical excision and has had stable disease 15 months after radiosurgical treatment with the linear accelerator (LINAC) system. In this setting, stereotactic radiosurgery provided a useful adjunct to thoracoscopic microsurgical resection. Future Class I and II evidence should be sought to evaluate the utility of stereotactic radiosurgery as a primary treatment modality or as an adjuvant for microneurosurgical resection of benign spinal lesions in patients who want noninvasive treatment after disease recurrence or who harbor medical comorbidities that would preclude them from being safe surgical candidates.

## 1. Introduction

Stereotactic radiosurgery has been an important addition to the armamentarium of treatment options available to patients with neurosurgical diseases. Its efficacy in the treatment of a host of benign and malignant intracranial lesions is well established, and it has been used with great frequency since the mid-1990s [1]. Stereotactic radiosurgery for the treatment of intracranial metastases with or without prior whole brain irradiation has been demonstrated to have a control rate of 85% to 95% [2, 3], and thus it provides patients a noninvasive means of disease palliation. Its role in the treatment of benign intracranial lesions such as arteriovenous malformations, meningiomas, vestibular schwannomas, and pituitary adenomas is also widely supported in the literature [4, 5]. The benefits from this noninvasive treatment modality have been achieved when it is used either as an adjunct to prior surgery or as a sole treatment modality in patients with both malignant and benign disease. 

The role of radiation therapy in the treatment of spinal column disease has been more limited than its use in intracranial lesions. More recently, technological advances in stereotactic radiosurgery have allowed precise targeting of the radiation beams for delivery of large doses of radiation sufficient to treat disease states without the adverse effects of radiation on the adjacent spinal cord. Data supporting the use of postoperative radiotherapy in subtotally resected spinal masses or recurrent disease are scarce. We report the case of a patient with a thoracic spinal schwannoma who had an interval increase of his lesion five months after operative resection. In this setting, stereotactic radiosurgery has provided a useful adjunct to thoracoscopic microsurgical resection in the setting of recurrent benign pathology of the spine [6, 7]. 

## 2. Case Report

**Figure 1:**
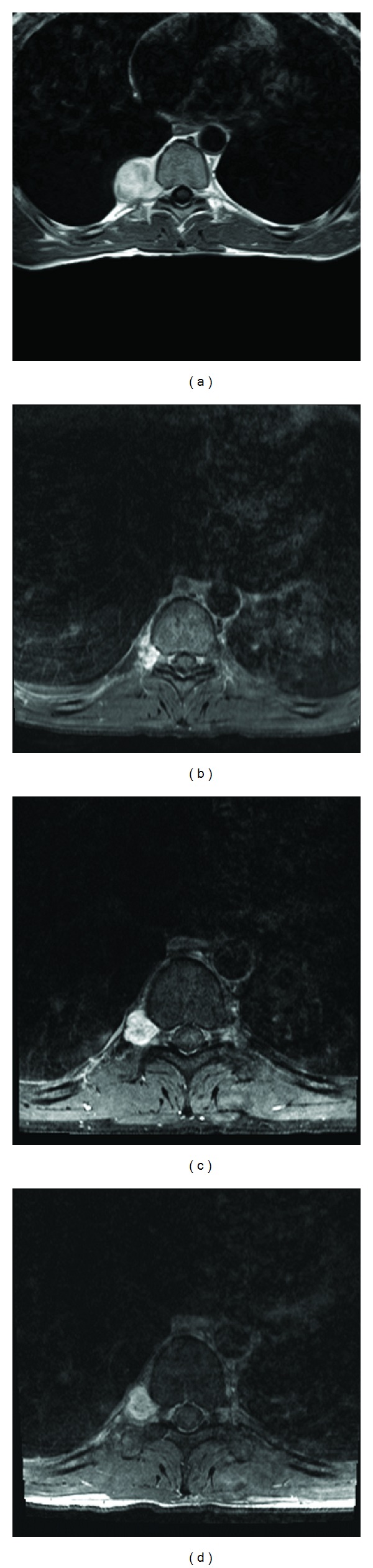
Magnetic resonance imaging of the thoracic spine with gadolinium contrast at the T9-10 region demonstrating an enhancing intradural extramedullary lesion exiting the neural foramen. (a) Preoperative MR imaging showing the initial size of the mass (3.8 × 3.6 × 4.5 cm) with no spinal cord compression. (b) Immediate postoperative MR image demonstrating subtotally resected mass with a 1.5 × 1.0 × 1.5 cm residual within the right neural foramen. (c) MR imaging obtained 5 months postoperatively demonstrating progression of the enhancing component measuring 1.9 × 1.0 × 1.7 cm. (d) MR imaging with gadolinium enhancement showing stable disease 15 months after stereotactic radiosurgery.

The patient is a 30-year-old man who had a two-year history of intermittent episodes of right-sided chest pain, numbness, and tingling in the T9 and T10 dermatomal distribution. The results of the remainder of his neurological examination were normal. MR imaging demonstrated a T9-T10 enhancing right-sided paraspinal mass (3.8 × 3.6 × 4.5 cm) invading the T9-T10 foramen with no spinal cord compression (Figure 1(a)). The patient had no prior history of any other lesions, and there was no family history of any phakomatoses. The patient underwent CT-guided needle biopsy of this lesion at an outside facility, and the results of the pathological analysis were consistent with a diagnosis of neurofibroma. The patient opted to forgo any further treatment, but episodes of numbness and tingling increased in frequency and the patient was then referred for neurosurgical intervention.

The patient was counseled with respect to the various treatment options, including further watchful waiting, right thoracoscopic microsurgical tumor resection, or posterior costotransversectomy. He was also counseled to the potential risk of having the lesion transform into a malignant peripheral nerve sheath tumor. The patient elected to undergo a right thoracoscopic tumor resection. A near-total resection was achieved, and postoperative MR imaging demonstrated a 1.5 × 1.0 × 1.5 cm residual within the right neural foramen (Figure 1(b)). Pathological analysis of the tissue sample revealed that it was a WHO Grade I schwannoma. The patient declined definitive tumor removal with a posterior costotransversectomy at this time. 

On follow-up imaging obtained five months postoperatively, there was an interval increase in the size of the mass to 1.9 × 1.0 × 1.7 cm (Figure 1(c)). After additional counseling regarding definitive operative resection, the patient declined further surgical intervention and elected to undergo single-fraction T9-T10 radiosurgery. He underwent stereotactic radiosurgery using the Novalis linear accelerator (LINAC) radiosurgery gantry using the Brainlab corset. A single target volume defined by a single isocenter and radiation therapy commenced, with the patient receiving 15 Gy (1500 cGy) to the 90th isodense line through the five arcs covering 95% of the tumor volume. This dosage plan was a collaborative approach developed between the neurosurgeons and the radiation oncologists. The dosage is an intermediate dosage chosen because it has been used in the past by us without any ill effects to the spinal cord. The patient tolerated this procedure with no adverse effects and 15 months after the procedure has had stable disease with no recurrence of symptoms (Figure 1(d)). 

## 3. Discussion

Standard treatment for benign spinal lesions has historically involved surgical extirpation of the disease with decompression of the surrounding neural structures with or without spinal fixation for stabilization of the spine. In selected cases, we use a thoracoscopic microsurgical approach for resection of thoracic spinal schwannomas [6, 7]. The prognosis of completely resected benign disease is good, and reresection is advocated for recurrence of disease. Radiation to benign tumors of the spine as a primary treatment modality has not been advocated, and descriptions of its role as an adjunct to neurosurgical resection are scarce in the literature [8]. 

Unlike conventional external beam radiotherapy, which delivers a full dose of radiation to the target lesion and surrounding radiosensitive spinal cord, stereotactic radiosurgery using the LINAC or CyberKnife system can deliver a high-dose single fraction of radiation to the target tissue and spare most of the adjacent spinal cord [8, 9]. This decreases the risk of the most feared complication of spinal radiation, spinal cord myelopathy, which can develop months to years after the initial exposure to radiation. 

Ryu et al. [9] used Cyberknife stereotactic radiosurgery at a dosage of 1100 to 2500 cGy in one to five fractions in a series of 16 patients with either malignant or benign spinal disease not previously resected. They found that the alignment of the treatment dose with the target volume was within ±1 mm by use of spine fiducials and the Cyberknife treatment planning system. Six months after the procedure, all patients had stable disease and no new neurological symptoms as a result of the radiation or progression of disease. Dodd and colleagues [10] retrospectively reviewed a series of 51 patients with benign intradural extramedullary spinal cord lesions. Total treatment dosage ranged from 1500 to 3000 cGy delivered in consecutive daily sessions in an effort to manage the patients nonoperatively. Three of the 51 patients required surgical resection of their lesion because of neurological worsening of their symptoms. Twenty-eight patients had a median followup of 36 months with either stable or decreased radiographic evidence of disease. Only one patient experienced radiation-induced toxicity of the spinal cord 8 months after treatment. 

Gerszten et al. [8] reported the use of Cyberknife in a series of 15 patients with benign spinal lesions who underwent single-fraction radiosurgery. The tumor dosage was 12–20 Gy to the 80% isodose line contoured at the edge of the target volume with the maximum intratumoral dose ranging from 15 to 25 Gy. No acute radiation-induced toxicity was seen in any of the patients treated, and no tumor progression was documented on follow-up imaging 12 months after the procedure. Gerszten and colleagues [11] also examined a larger series of patients with benign intradural extramedullary lesions treated with stereotactic radiosurgery. Seventy-three patients with benign intradural extramedullary lesions were included in the series, 19 of whom had undergone prior surgical resection. The maximum intratumor dosage was 2500 cGy. The authors demonstrated long-term radiographic tumor control in all patients (median follow-up 37 months); however, three patients in the series experienced symptoms consistent with radiation-induced toxicity of the spinal cord 5 to 13 months after treatment. 

More recently, Chang and colleagues [12] retrospectively evaluated a series of 20 patients with 30 benign intradural spinal tumors, both intra- and extramedullary. Radiosurgery was used as primary treatment in 22 lesions, for postoperative tumor control in 4 lesions and for image-based progression in the remaining 4 lesions. A 1400- to 3300-cGy marginal dose was delivered in 1–5 fractions. Median follow-up of 35.6 months demonstrated that 57% of lesions decreased in size, 33% remained unchanged, one increased without response, and two initially decreased and then increased in size. Three patients developed symptoms consistent with radiation-induced toxicity of the spinal cord. Murovic and colleagues [13] retrospectively reviewed outcomes in a group of 15 patients with 18 foraminal tumors treated with dosages of 1600 to 2400 cGy. Tumor volumes decreased in 67% of patients (8/10 schwannomas and 3/7 neurofibromas). Selch et al. [14] also retrospectively reviewed a group of 25 patients with benign nerve sheath tumors that were treated with stereotactic radiation (median peripheral dosage 1200 cGy). Tumor size remained stable in 18 cases, and 28% of patients had reduction in the size of their tumors. There was no evidence of radiation-induced toxicity of the spinal cord. Optimal radiation doses to the spinal column using a single fraction of radiation have not been investigated, and therefore long-term consequence of radiation exposure in this fashion is not well known. Instead, the dose to the tumor margin was dictated by tumor histology, location, and history of prior fractionated radiotherapy in the previously mentioned cases studies [8, 9].

Our case is a valuable addition to the literature in that we used adjuvant radiation after thoracoscopic microsurgical resection of a benign spinal schwannoma that had exhibited early evidence of growth. After stereotactic radiosurgery, the patient's disease remained stable after 15 months and he experienced no neurological sequelae from treatment. Future Class I or II studies should be performed to evaluate the utility of stereotactic radiosurgery as a primary treatment modality in benign spinal lesions or as an adjuvant for microneurosurgical resection in those patients who want noninvasive treatment after evidence of recurrence of disease or who harbor medical comorbidities that would preclude them from being appropriate surgical candidates. Stereotactic radiosurgery has been shown to be successful in patients harboring vestibular schwannomas as a primary treatment modality or as adjuvant treatment after microneurosurgical resection. Given the similarity in the pathology of spinal schwannomas and vestibular schwannomas, with location being the key differentiating factor, this modality may also have greater utility in the treatment of spinal schwannomas.
